# Editorial: Biomaterials used in tissue engineering for the restoration of ocular disorders

**DOI:** 10.3389/fphar.2024.1369505

**Published:** 2024-01-31

**Authors:** Luis M. Rodríguez-Lorenzo, Felisa Reyes-Ortega, May Griffith

**Affiliations:** ^1^ Department Polymeric Nanomaterials and Biomaterials, Institute of Polymer Science and Technology (ICTP), Madrid, Spain; ^2^ Department Ophthalmology, Maimonides Biomedical Research Institute of Cordoba (IMIBIC), Reina Sofia University Hospital and University of Cordoba, Cordoba, Spain; ^3^ Maisonneuve-Rosemont Hospital Research Centre, Montreal, QC, Canada; ^4^ Department Ophthalmology, Université de Montréal, Montreal, QC, Canada; ^5^ Institute of Biomedical Engineering, Université de Montréal, Montreal, QC, Canada

**Keywords:** ocular disorders, 3D bioprinting, short peptide, clinical trial, cornea, biosealants, fillers, decellularized tissues

The goal of tissue engineering is the construction of bioartificial tissues to restore, improve, or maintain failing or failed tissue and organs. The concept was born and is still driven by the necessity of addressing the critical gap between the growing number of patients awaiting tissue or organ transplantation and the severe shortfall of donated organs/tissues (Chandra et al., 2020). Tissue engineering has been very broadly described to use cells, scaffolds, and tissue-inducing factors (Langer and Vacanti, 1993), which translate to the use of biomaterials, bioengineering, and associated regenerative medicine technologies.

Ocular disorders affect a large percentage of the world population: at least 1 billion people have vision impairment that could have been prevented or has yet to be addressed. Vision impairment impacts the lives of people everywhere and affects people of all ages, with the majority being over the age of 50 (World Health Organization, 2019). Tissue engineering is a promising tool to produce treatments for ophthalmologic disorders; however, several obstacles need to be overcome.

The required range of properties for biomaterials to be used in the restoration of the eye from damage by ocular disorders is limited by the optimal combination of thickness, mechanical properties, and composition, ensuring bio- and immune-compatibility. Ideally, the biomaterials should resist the build-up of proteins and other substances that would hinder the intended function, and they should be resistant to infection. The ultimate challenge is the capacity to surgically implant the engineered tissue with no complications for the patient.

From a biomaterials standpoint, the first-in-human report of biomimetic material engineered implants that stimulated stable regeneration of human corneal tissues and nerves was published just over a decade ago. Griffith, Fagerholm, and their team used cell-free recombinant human collagen-based implants to discover that the adult human cornea was capable of regeneration by inducing the patients’ endogenous cells to make the repairs in an early clinical trial (Fagerholm et al., 2010). More recent developments come from advanced manufacturing technologies. 3D printing and bioprinting are becoming the technologies of choice for the manufacture of scaffolds and constructs in tissue engineering because they enable the design of personalized tissue and organ constructs for precision medicine. The current challenge is to find the right combination of printable materials and printing technology. There is growing use of decellularized tissues as a base for the printable ink to provide an adequate environment for recellularization with healthy biological material. However, not only do recellularization techniques need to be optimized, but also the most suitable therapeutic cells, from pluripotent stem cells (iPSCs) to human embryonic stem cells (hESCs). An alternate approach has been the development of fully synthetic mimetic macromolecules that can be designed to be fully chemically controlled and safe from pathogen transmission, as well as being cost effective as there is no need for expensive screening to minimize the risk of transmissible pathogens, particularly zoonotic transmission. For example, short peptide mimics of full-length proteins have been tested for tissue restoration (Rubert Pérez et al., 2015).

As alternatives to fully engineered ocular scaffolds or implants, biomaterials have been tested as pro-regeneration sealants and fillers. These biosealant fillers could be either the hydrogel matrices based on collagen-like peptides and polyethylene glycols, mixed with fibrinogen to promote adhesion within tissue defects (McTiernan et al., 2020) or chemically modified gelatin with polymethacrylates, like GeIMA (Yan et al., 2022), GELCORE (Sani et al., 2019), or GELGYM (Sharifi et al., 2021 **)**. They are less costly and reduce any risk of allergy or immune rejection associated with xenogeneic materials.

The current Research Topic covers cutting-edge research on several aspects of ocular tissue engineering mentioned above. For Fuchs endothelial corneal dystrophy (FECD), the characteristics of the different biomaterials proposed for the engineering of a monolayer of corneal endothelium for the treatment of FECD, and a retinal pigment epithelium (RPE) monolayer for the treatment of dry age macular degeneration (AMD), are reviewed. It is noteworthy to read the reasons for not reaching ample clinical usage and the distance of the described properties from the clinical needs (Sasseville et al.). How highly effective modulator therapies (HEMT) targeting cystic fibrosis transmembrane conductance regulator (CFTR) protein influence the dynamic and interdependent processes of healthy and abnormal lens development during pregnancy and breastfeeding is also analyzed, highlighting the need for further research that clarifies the potential secondary effects of these therapies (Zhu et al.). The need for tissue engineered approaches for the treatment of corneal opacity is also covered, including a proposal for a new model of keratoprostheses based on the combination of 3D printing and decellularization techniques, where decellularized corneal tissue is used as the base material for the 3D ink (Wang et al.). In the same direction, a high-throughput strategy for designing a simplified, inexpensive, and scalable corneal xenograft platform is presented. The advantages and limitations of this decellularization technique are described (Wang et al.). The introduction of new approaches for healing corneal tissue is also included in this Research Topic. Corneal alkali burns are usually treated with corticosteroids; however, the side effects of these treatments advocate the continued search for alternatives. The paper (Thathapudi et al.) proposes and describes the potential of an alternative based on a cannabinoid receptor 2 (CB2r) agonist that is effective but extremely insoluble, necessitating the use of biomaterials as micelles for drug delivery to restore tissue morphology and function.
